# Supplementary figures and images for: Administration of novobiocin and apomorphine mitigates cholera toxin mediated cellular toxicity: Lessons from cholera toxin yeast model system
Source: PLoS One. 2024 Dec 5;19(12):e0315052. doi: 10.1371/journal.pone.0315052 (PMC11620602; doi:10.1371/journal.pone.0315052)

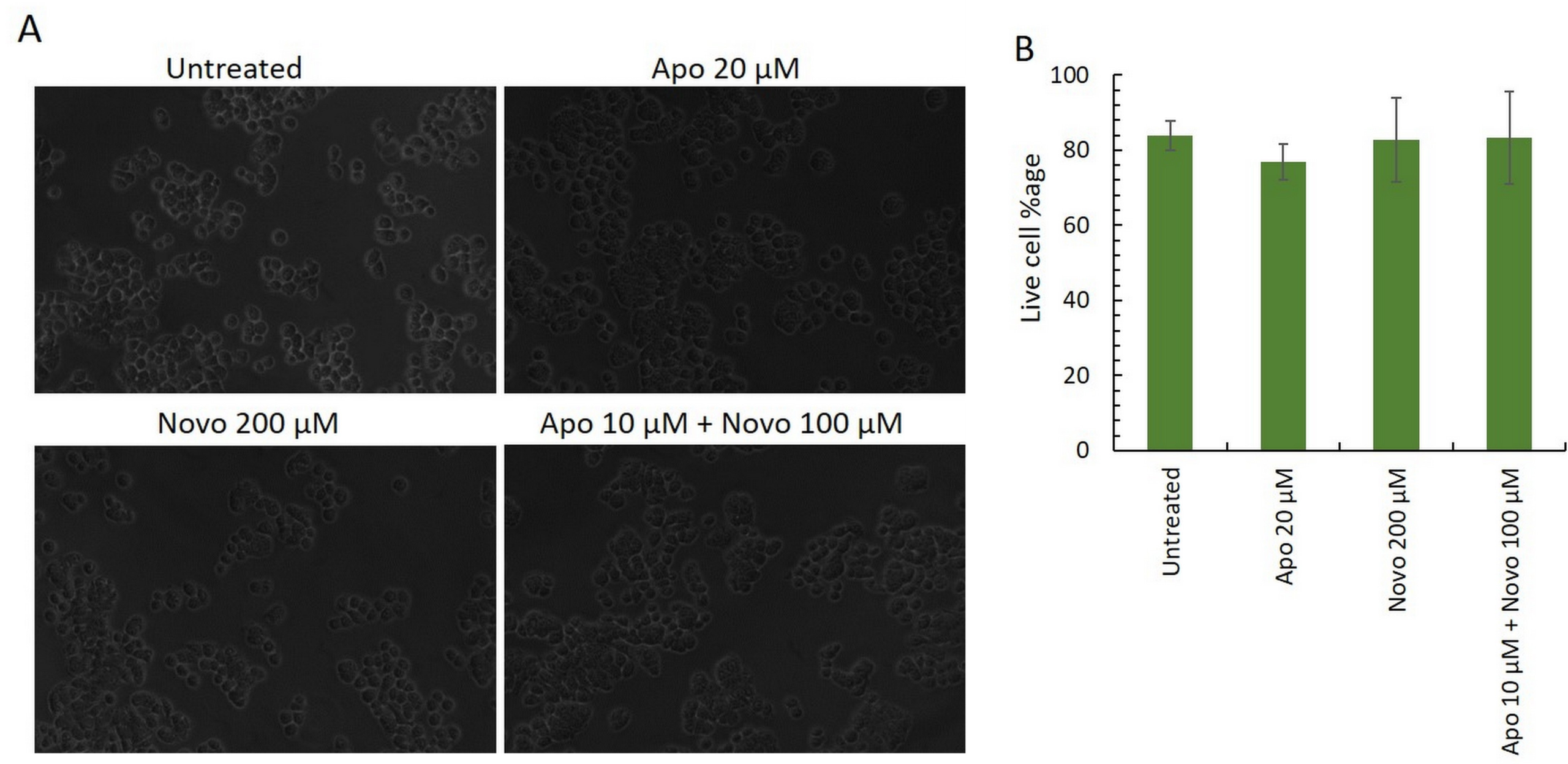

Supplement: S1 Fig — 0.2 million HT29 cells were seeded per well. At 60% confluence, cells were washed 2X with DPBS. Drugs treatment was given for 6 hrs. Images were taken at 20X magnification under a light microscope. B. Flow cytometry analysis: Comparison between cell viability after 24 hrs of drugs treatment. The data shown here is collected from two independent experiments. (TIF) [file pone.0315052.s001.tif]

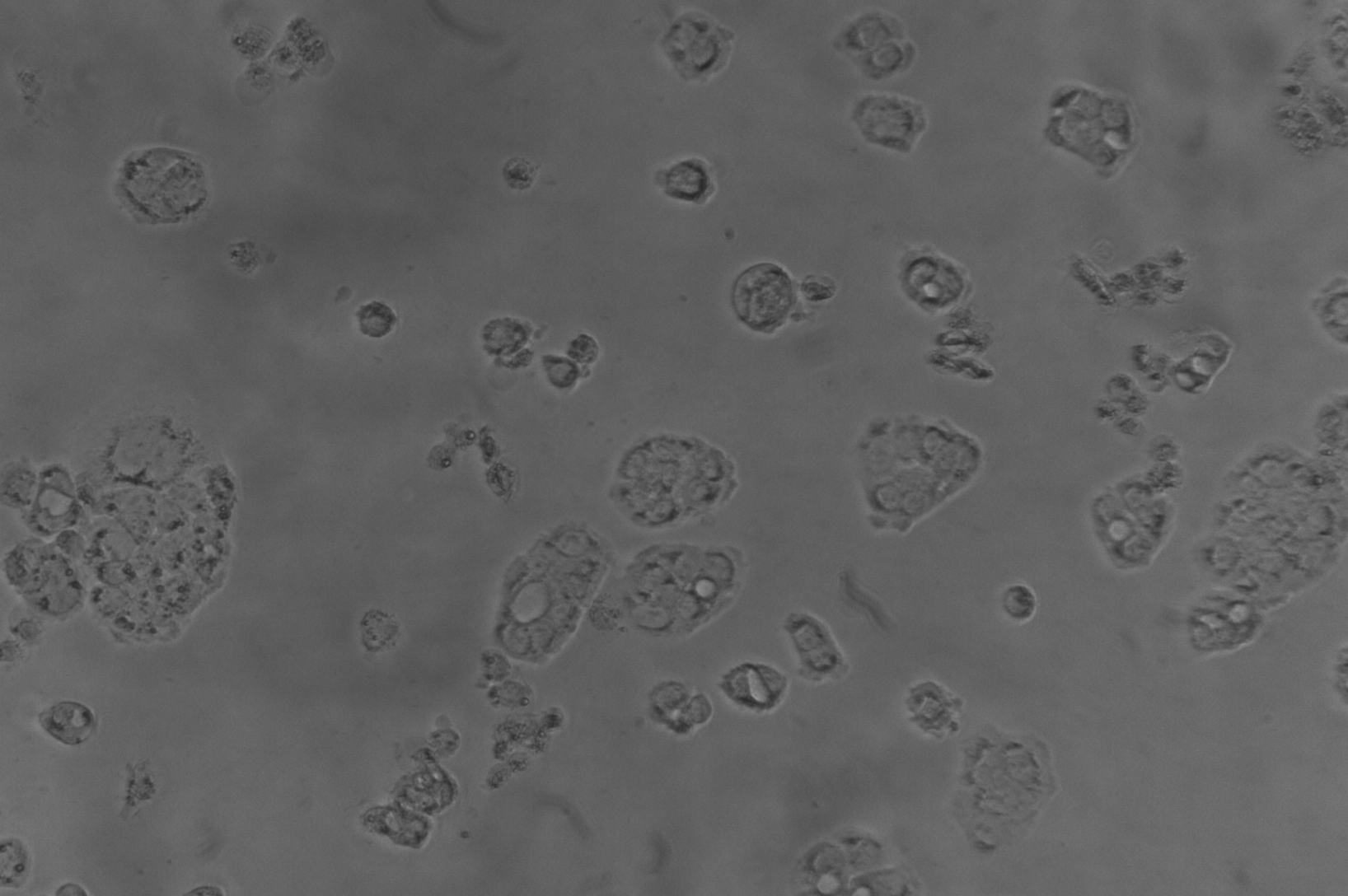

Supplement: S1 Data set — (ZIP) [file pone.0315052.s004.zip › minimal_data_set/Fig 4_Microscopy_Raw/CT 10.tif]

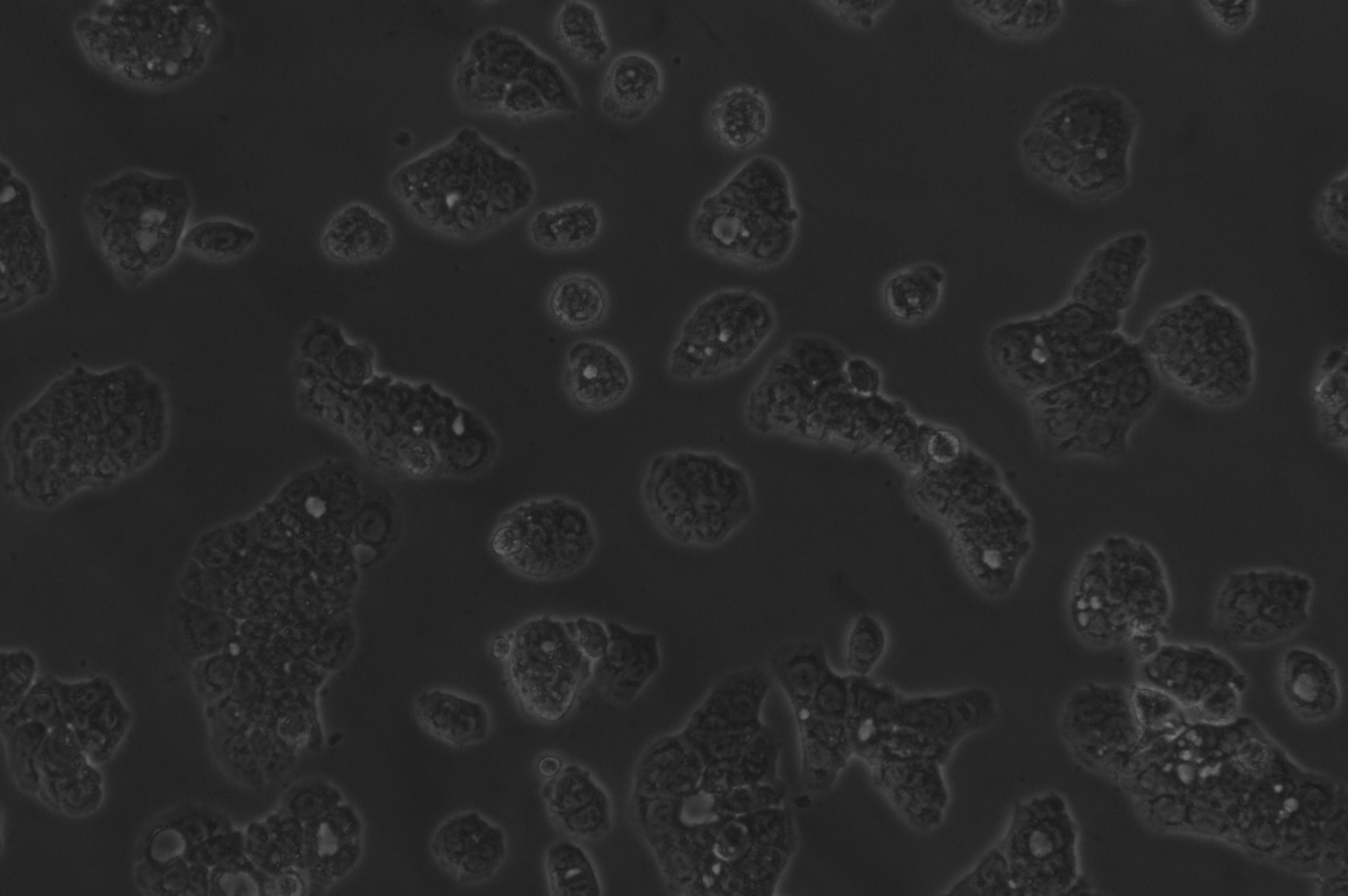

Supplement: S1 Data set — (ZIP) [file pone.0315052.s004.zip › minimal_data_set/Fig 4_Microscopy_Raw/CT10+ A10.tif]

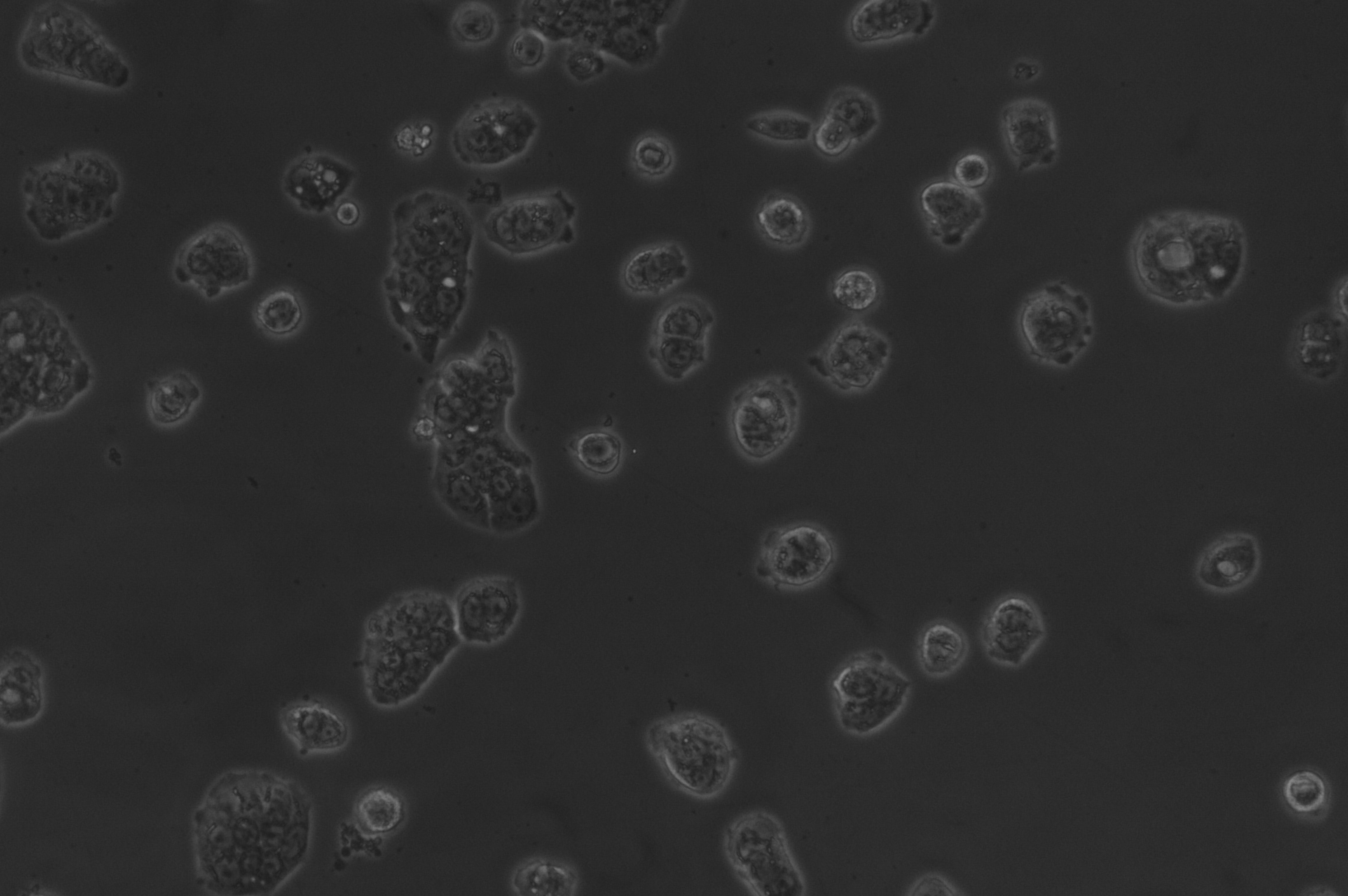

Supplement: S1 Data set — (ZIP) [file pone.0315052.s004.zip › minimal_data_set/Fig 4_Microscopy_Raw/CT10+A10+N100.tif]

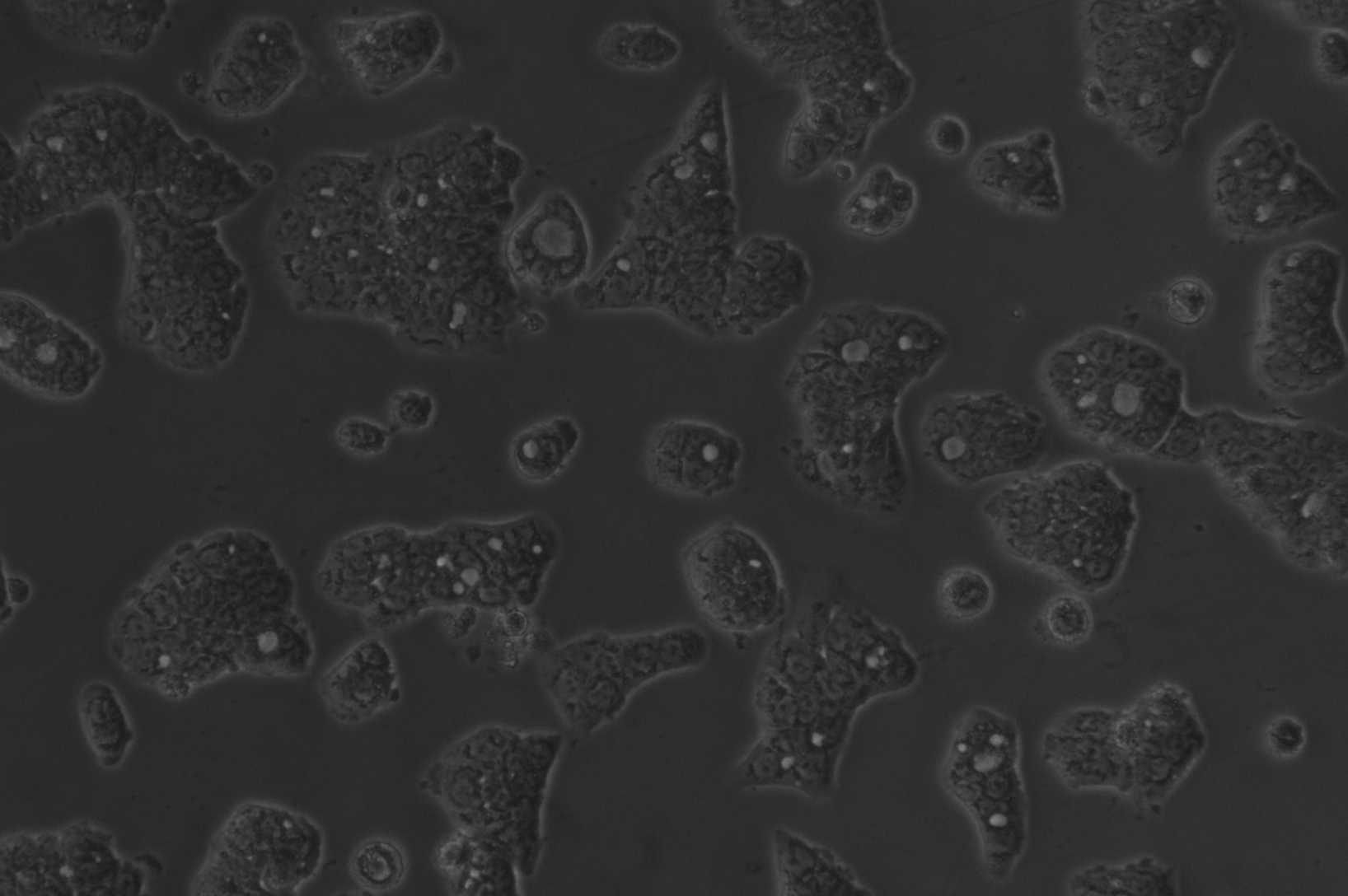

Supplement: S1 Data set — (ZIP) [file pone.0315052.s004.zip › minimal_data_set/Fig 4_Microscopy_Raw/CT10+A20.tif]

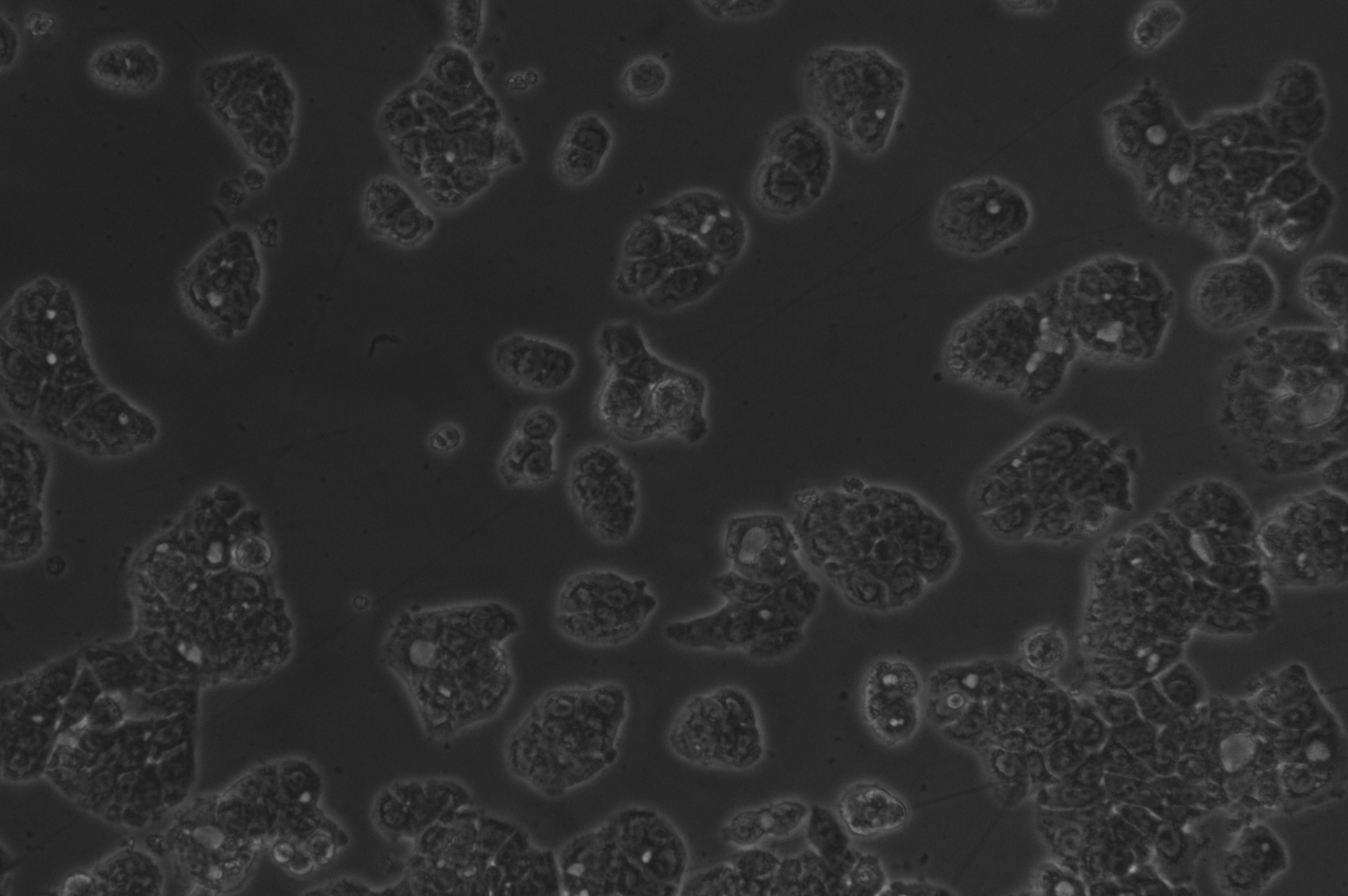

Supplement: S1 Data set — (ZIP) [file pone.0315052.s004.zip › minimal_data_set/Fig 4_Microscopy_Raw/CT10+N100.tif]

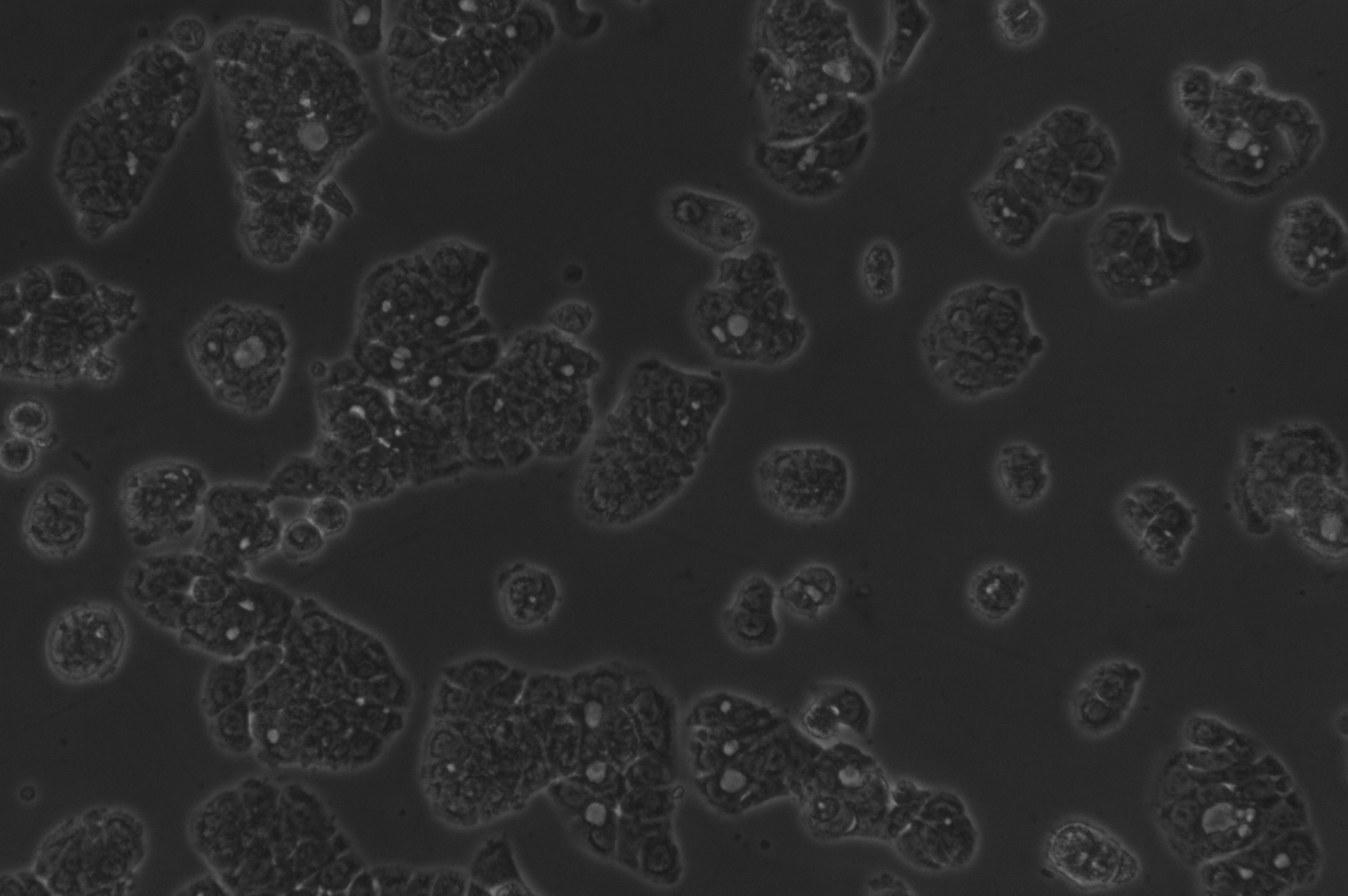

Supplement: S1 Data set — (ZIP) [file pone.0315052.s004.zip › minimal_data_set/Fig 4_Microscopy_Raw/CT10+N200.tif]

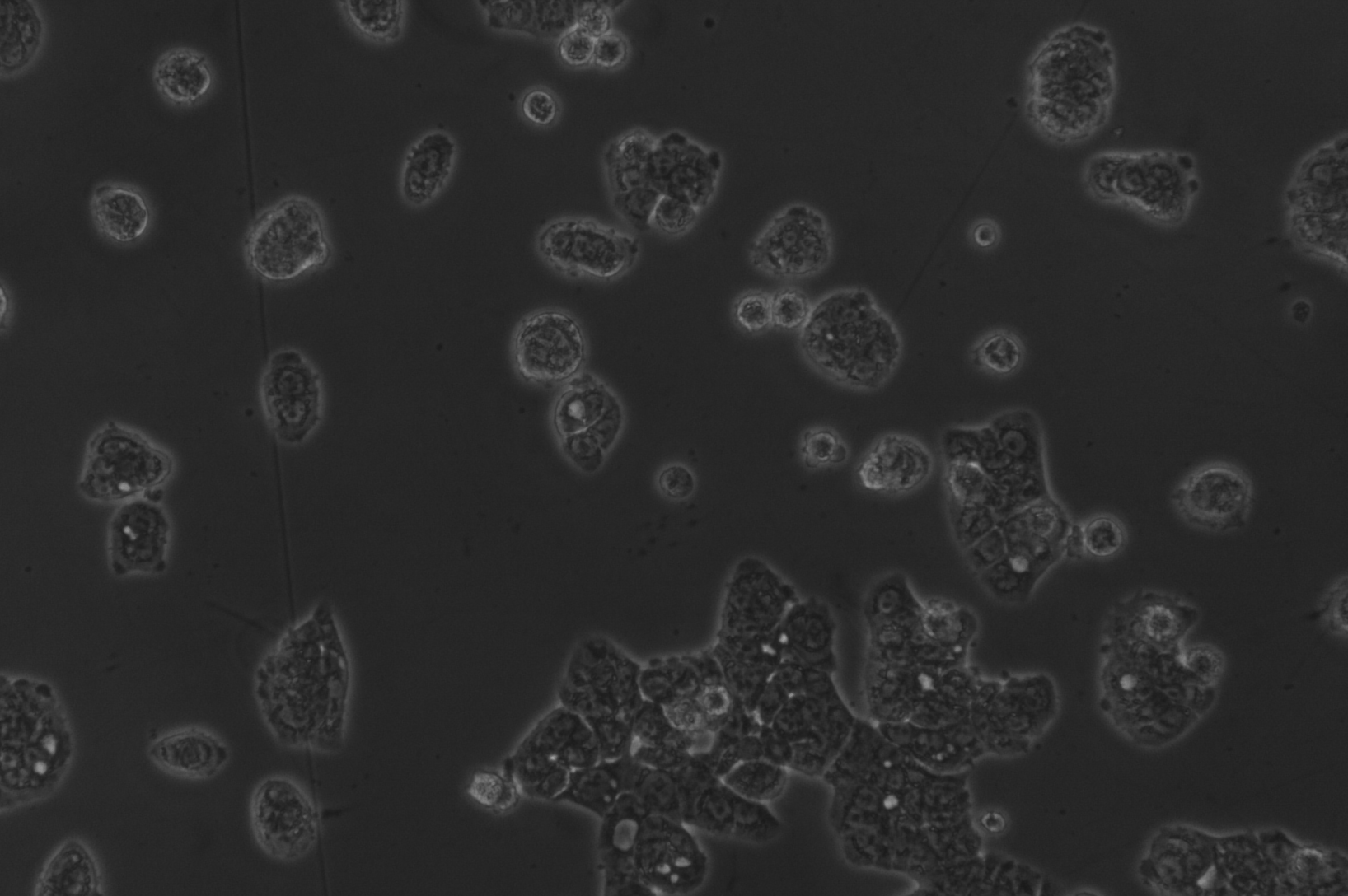

Supplement: S1 Data set — (ZIP) [file pone.0315052.s004.zip › minimal_data_set/Fig 4_Microscopy_Raw/CT40+A10+N100.tif]

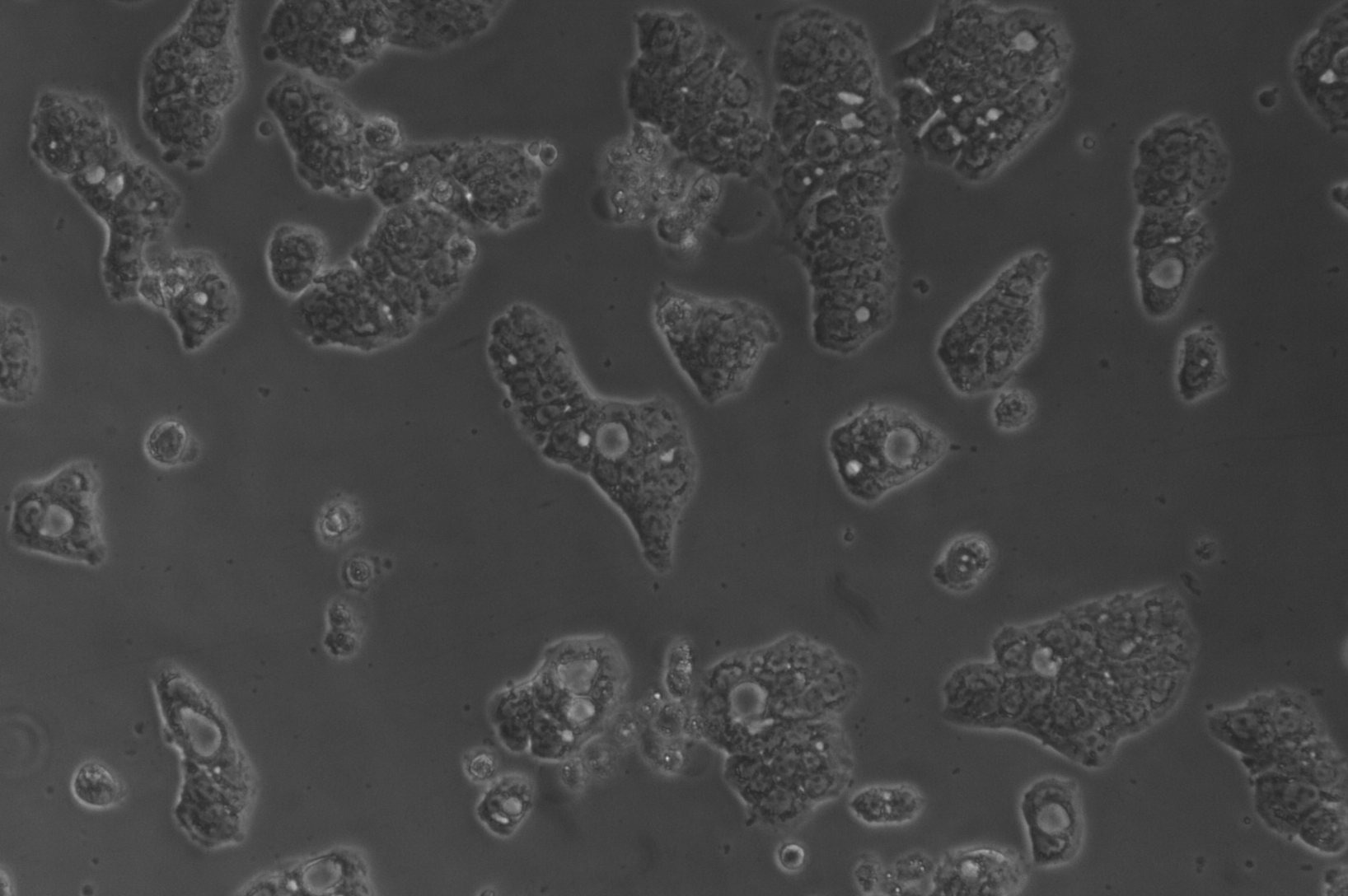

Supplement: S1 Data set — (ZIP) [file pone.0315052.s004.zip › minimal_data_set/Fig 4_Microscopy_Raw/CT40+A10.tif]

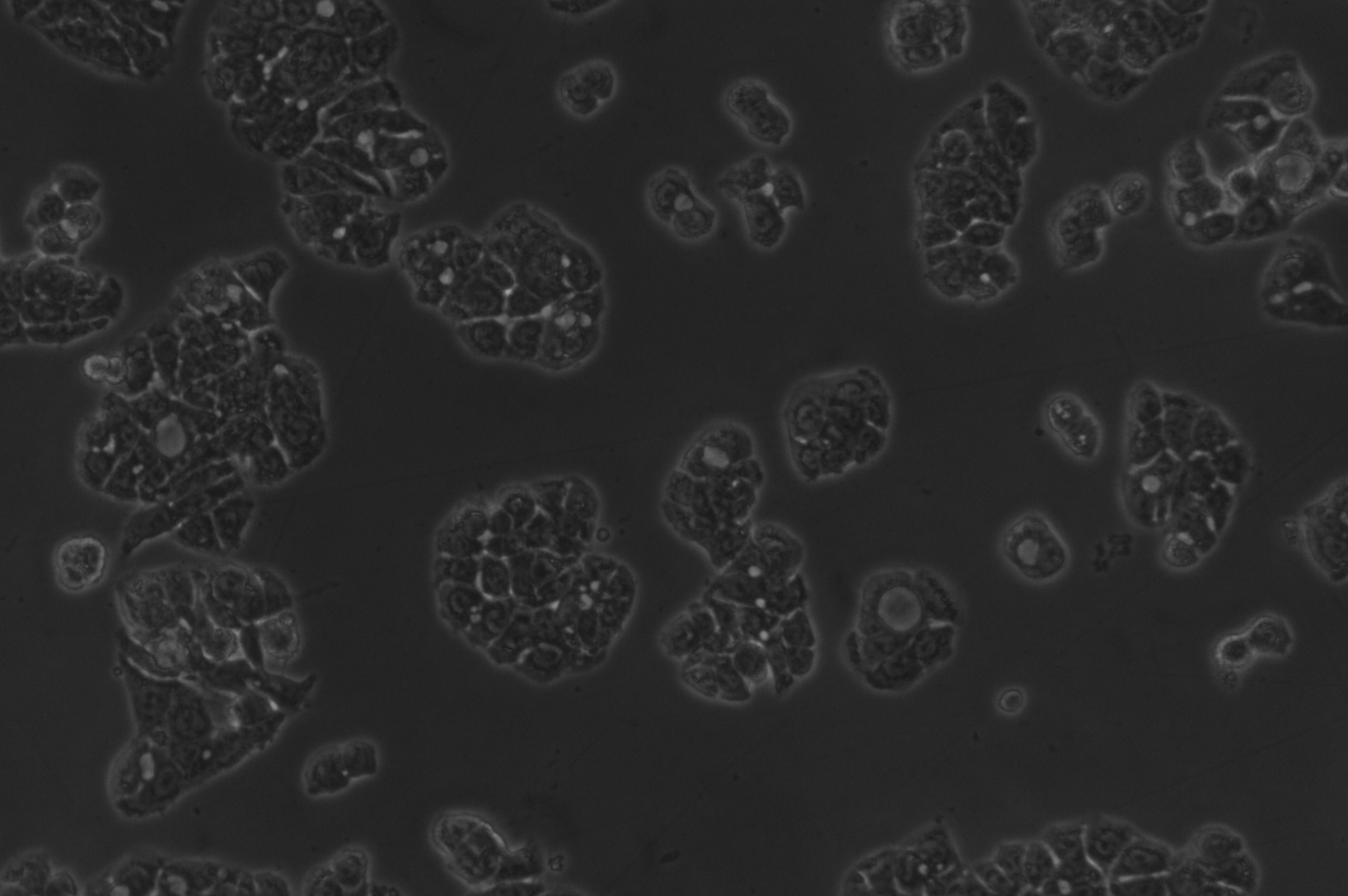

Supplement: S1 Data set — (ZIP) [file pone.0315052.s004.zip › minimal_data_set/Fig 4_Microscopy_Raw/CT40+A20.tif]

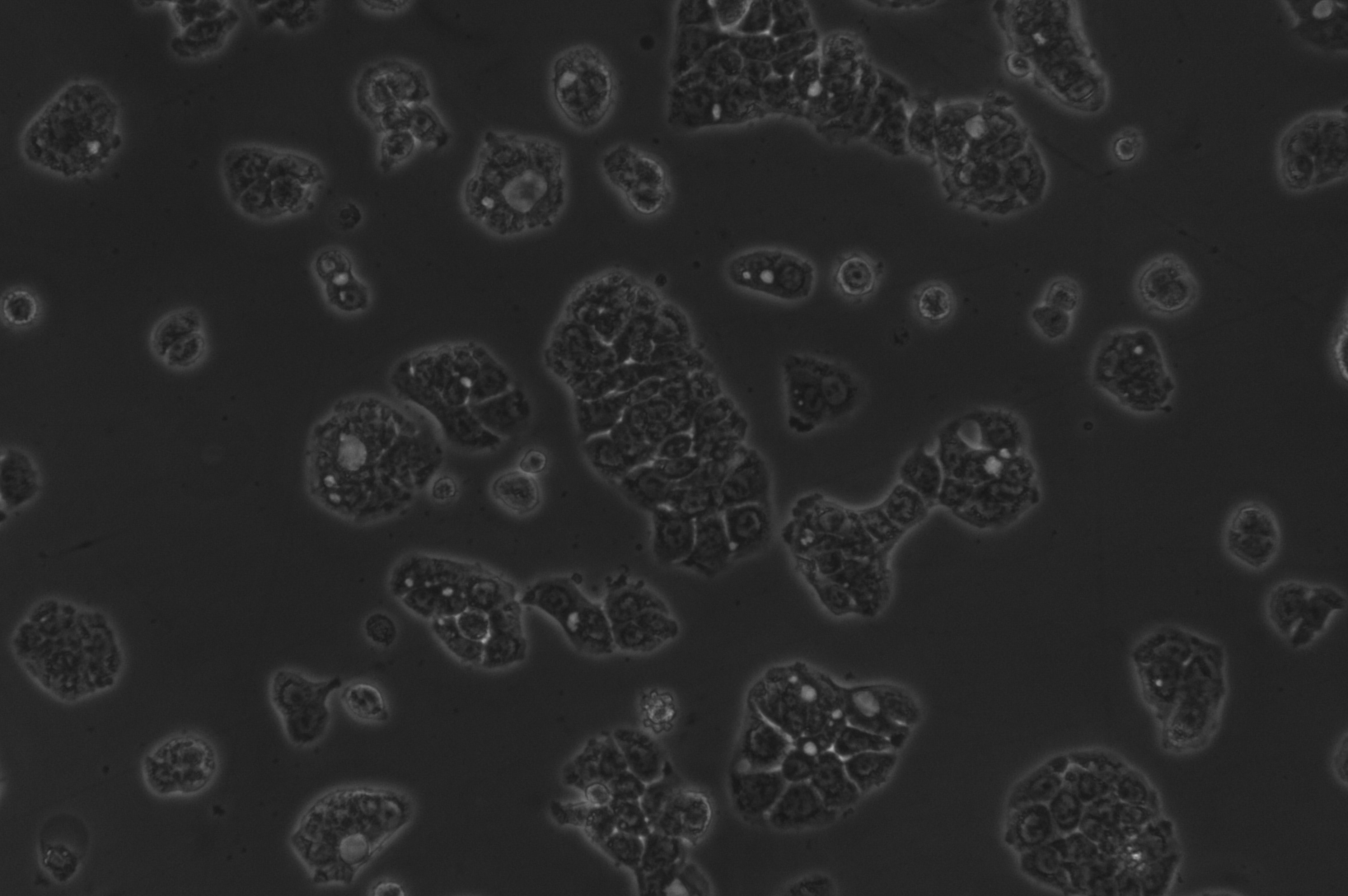

Supplement: S1 Data set — (ZIP) [file pone.0315052.s004.zip › minimal_data_set/Fig 4_Microscopy_Raw/CT40+N100.tif]

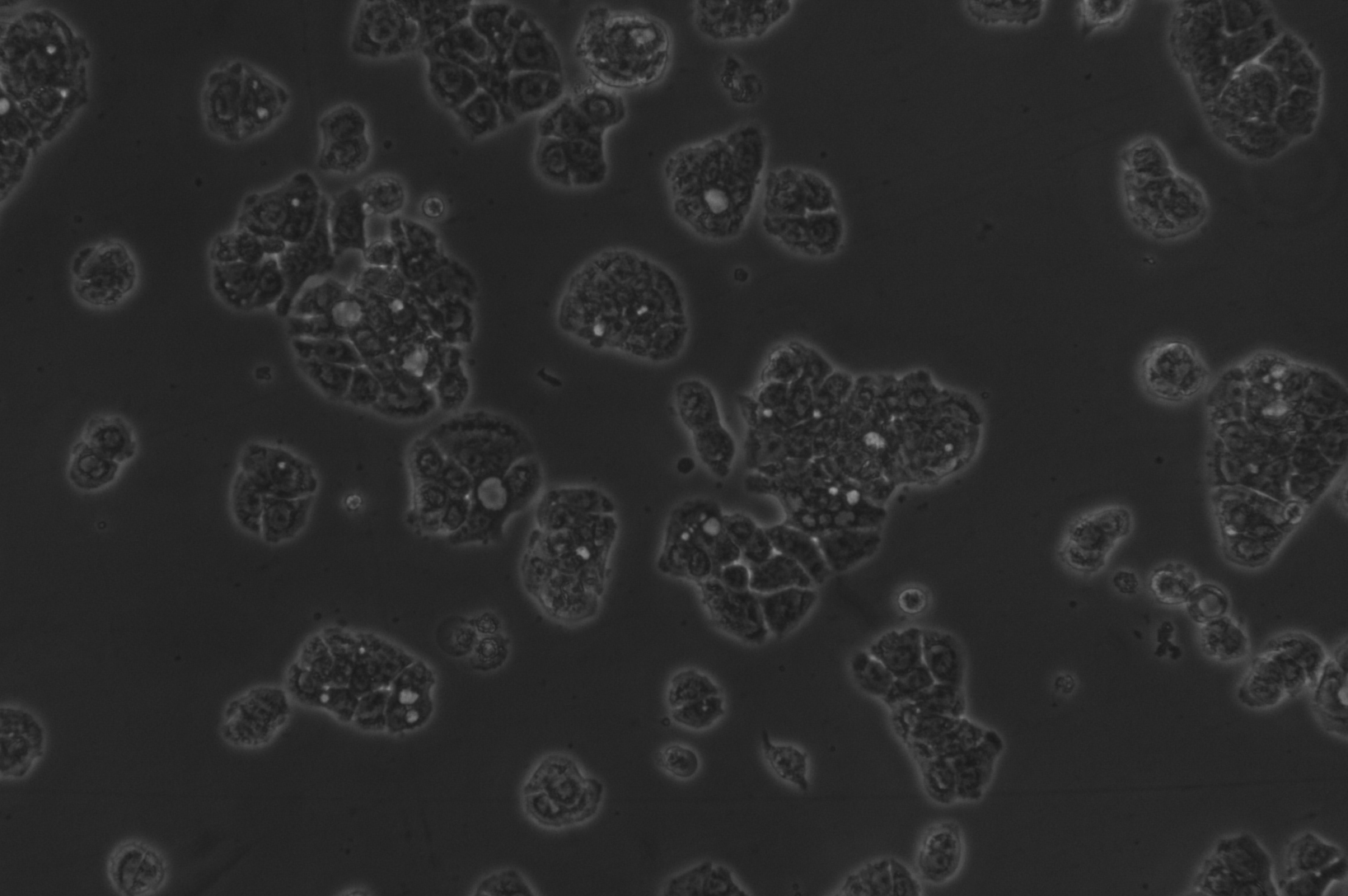

Supplement: S1 Data set — (ZIP) [file pone.0315052.s004.zip › minimal_data_set/Fig 4_Microscopy_Raw/CT40+N200.tif]

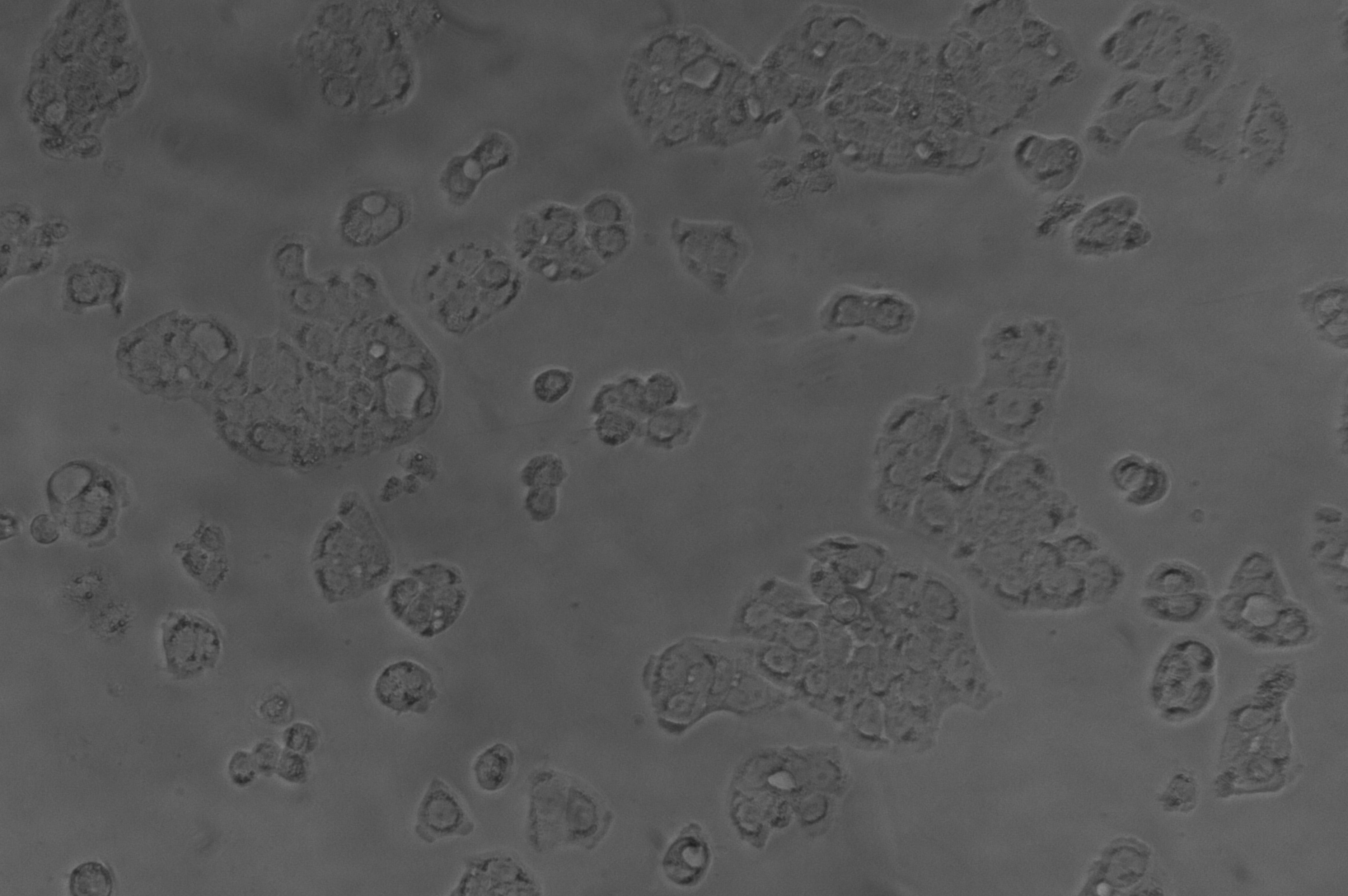

Supplement: S1 Data set — (ZIP) [file pone.0315052.s004.zip › minimal_data_set/Fig 4_Microscopy_Raw/CT40.tif]

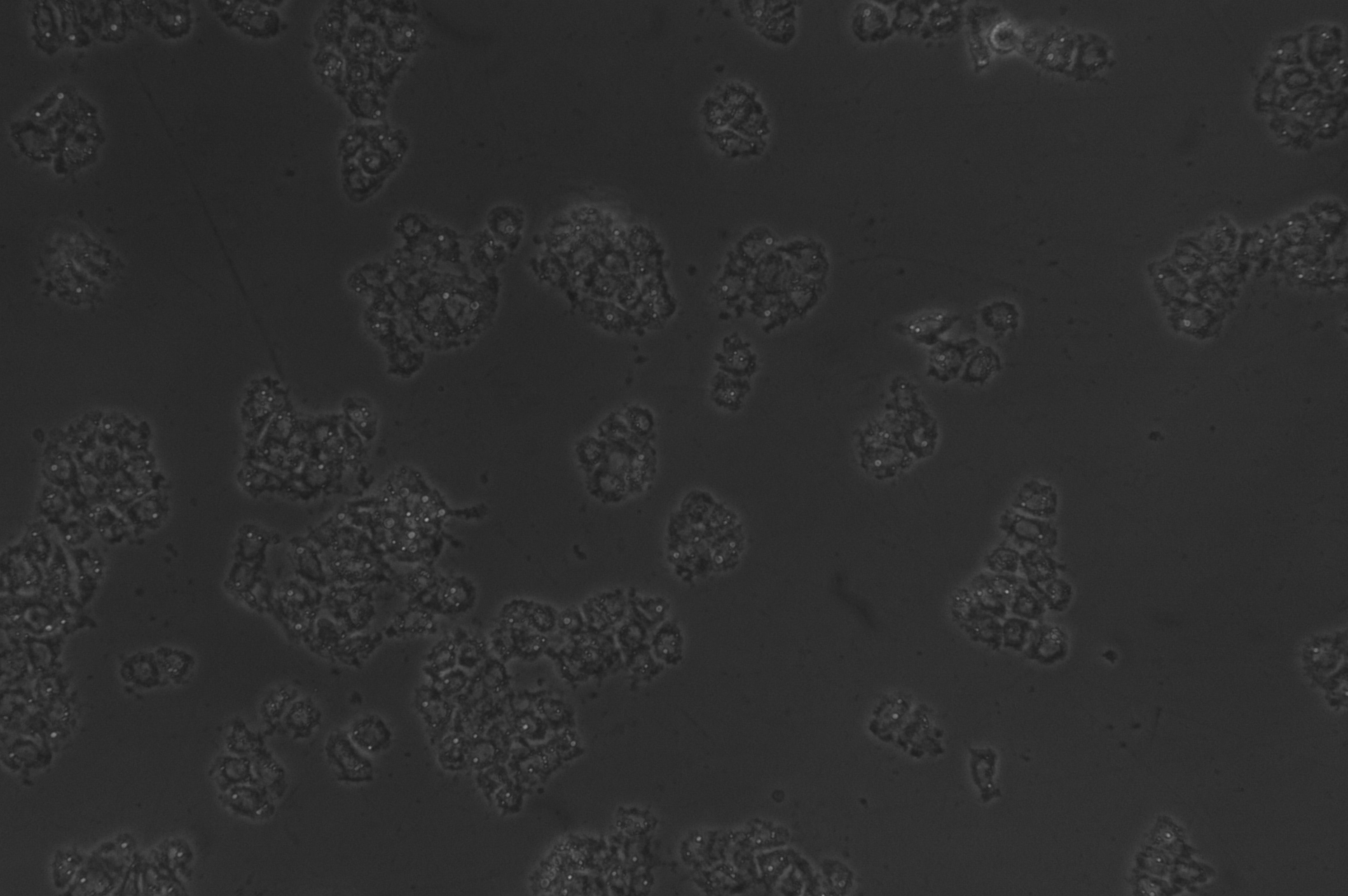

Supplement: S1 Data set — (ZIP) [file pone.0315052.s004.zip › minimal_data_set/Fig 4_Microscopy_Raw/Untreated.tif]
