# Supplementary material for: Administration of novobiocin and apomorphine mitigates cholera toxin mediated cellular toxicity: Lessons from cholera toxin yeast model system
Source: PLoS One. 2024 Dec 5;19(12):e0315052. doi: 10.1371/journal.pone.0315052 (PMC11620602; doi:10.1371/journal.pone.0315052)
Supplement: S1 Table — (DOC) [file pone.0315052.s002.doc]

| **Bacterial and yeast strains and cell lines** | | |
| --- | --- | --- |
| ***E. coli* DH5α** | Novagen | 70181 |
| **BY4741** | Bankapalli *et al., 2015, Bankapalli et al., 2017* | N/A |
| **HT29** | Gift from Dr Ravi Mishra, CSIR-IMTECH, INDIA | ATCC HTB-38 |
| **Plasmids** | | |
| **pESC-Leu** | Agilent technologies | #217452 |
| **pGML10** | RIKEN | RDB01957 |
| **HO-pGal-polyKanMX4-HO** | Addgene | #51664 |

**S1 table. Strains and plasmids used in the study**
