# Supplementary material for: Administration of novobiocin and apomorphine mitigates cholera toxin mediated cellular toxicity: Lessons from cholera toxin yeast model system
Source: PLoS One. 2024 Dec 5;19(12):e0315052. doi: 10.1371/journal.pone.0315052 (PMC11620602; doi:10.1371/journal.pone.0315052)
Supplement: S2 Table — (DOCX) [file pone.0315052.s003.docx]

**S2 table. Primers used in the study**

| **Primer name** | **SEQUENCE** |
| --- | --- |
| **XhoI/*ctxA* (pESC-Leu)(FP)** | 5’- CCG CTC GAG ATG AAT GAT GAT AAG TTA TAT C - 3’ |
| **NheI/*ctxA*  (pESC-Leu) (RP)** | 5’ - CTA GCT AGC TCA TAA TTC ATC CTT AAT TC- 3’ |
| **BamHI/ *ctxA* (pGML10) (FP)** | 5’- CGC GGA TCC ATG AAT GAT GAT AAG TTA TAT C - 3’ |
| **EcoRI /*ctxA* (pGML10) (RP)** | 5’- CCG GAA TTC TCA TAA CTC ATC CTT AAT TCT -3’ |
| **BamHI/ *ctxA* (HO-Locus) (FP)** | 5’- CGC GGA TCC ATG AAT GAT GAT AAG TTA TAT C -3’ |
| **SmaI /*ctxA* (HO-Locus)(RP)** | 5’- TCC CCC GGG TCA TAA CTC ATC CTT AAT TCT - 3’ |
