# Supplementary material for: Administration of novobiocin and apomorphine mitigates cholera toxin mediated cellular toxicity: Lessons from cholera toxin yeast model system
Source: PLoS One. 2024 Dec 5;19(12):e0315052. doi: 10.1371/journal.pone.0315052 (PMC11620602; doi:10.1371/journal.pone.0315052)

White ‘ **{** ‘ in some images show the region used for the final figures.

Minimal data set (Uncropped images)

Figure 1A

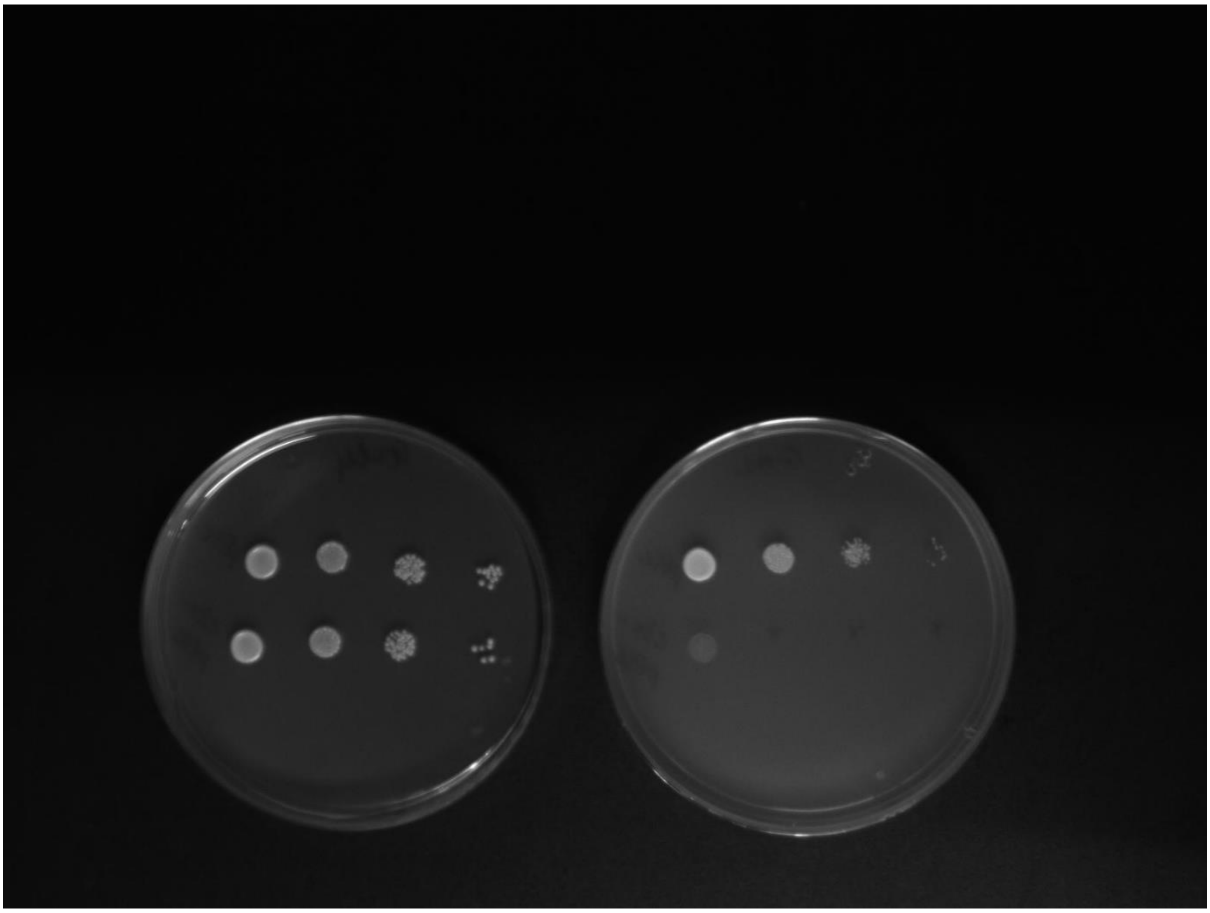

Figure 1C

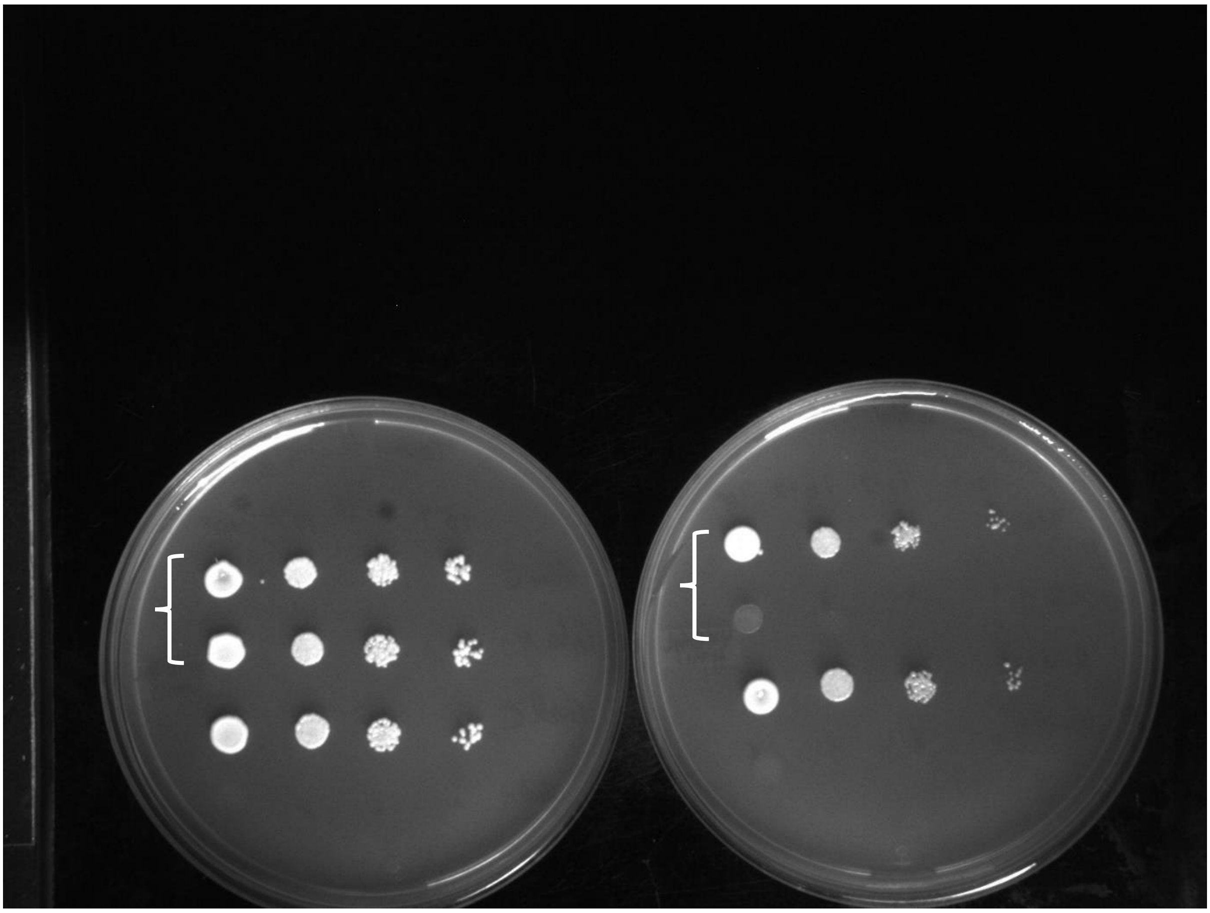

Figure 1E

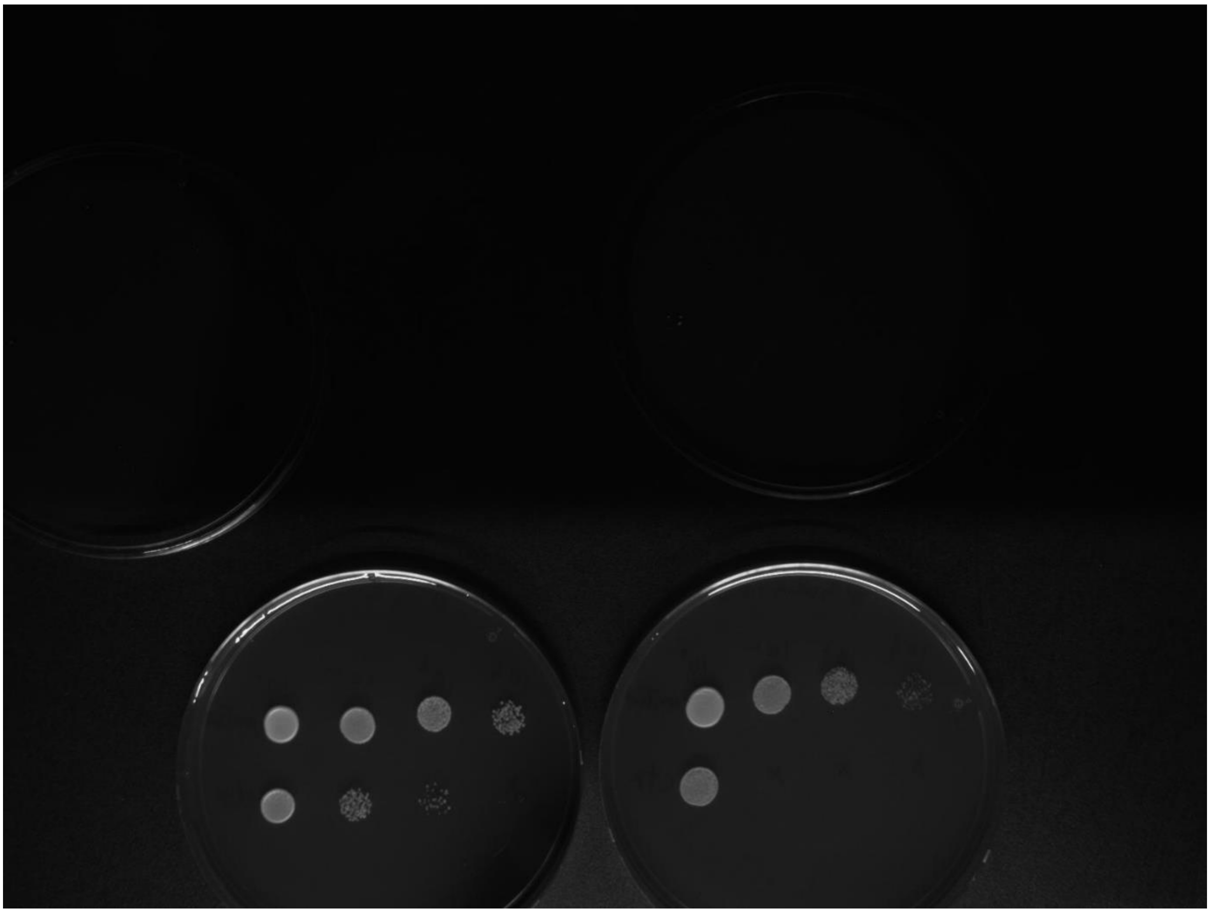

Figure 2C

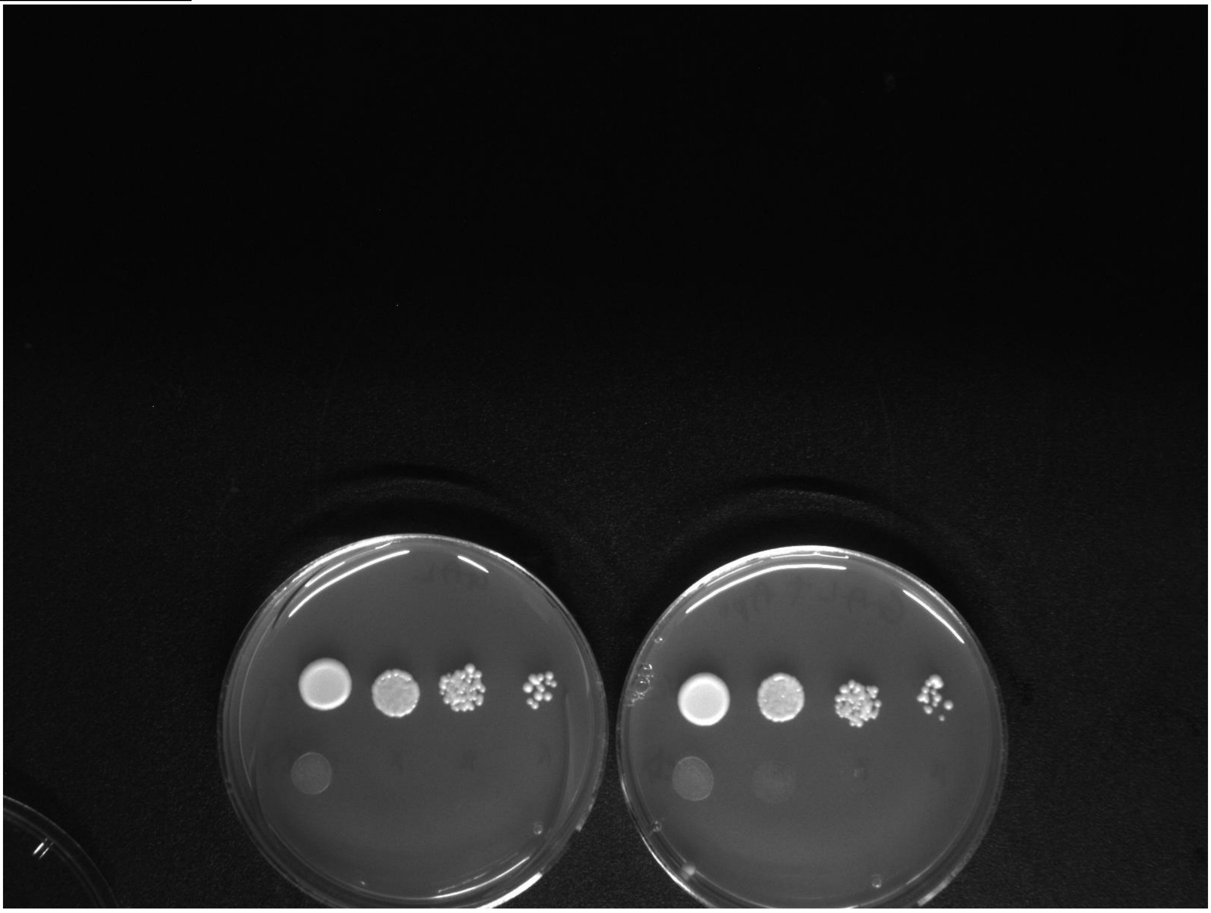

Figure 2D Upper panel

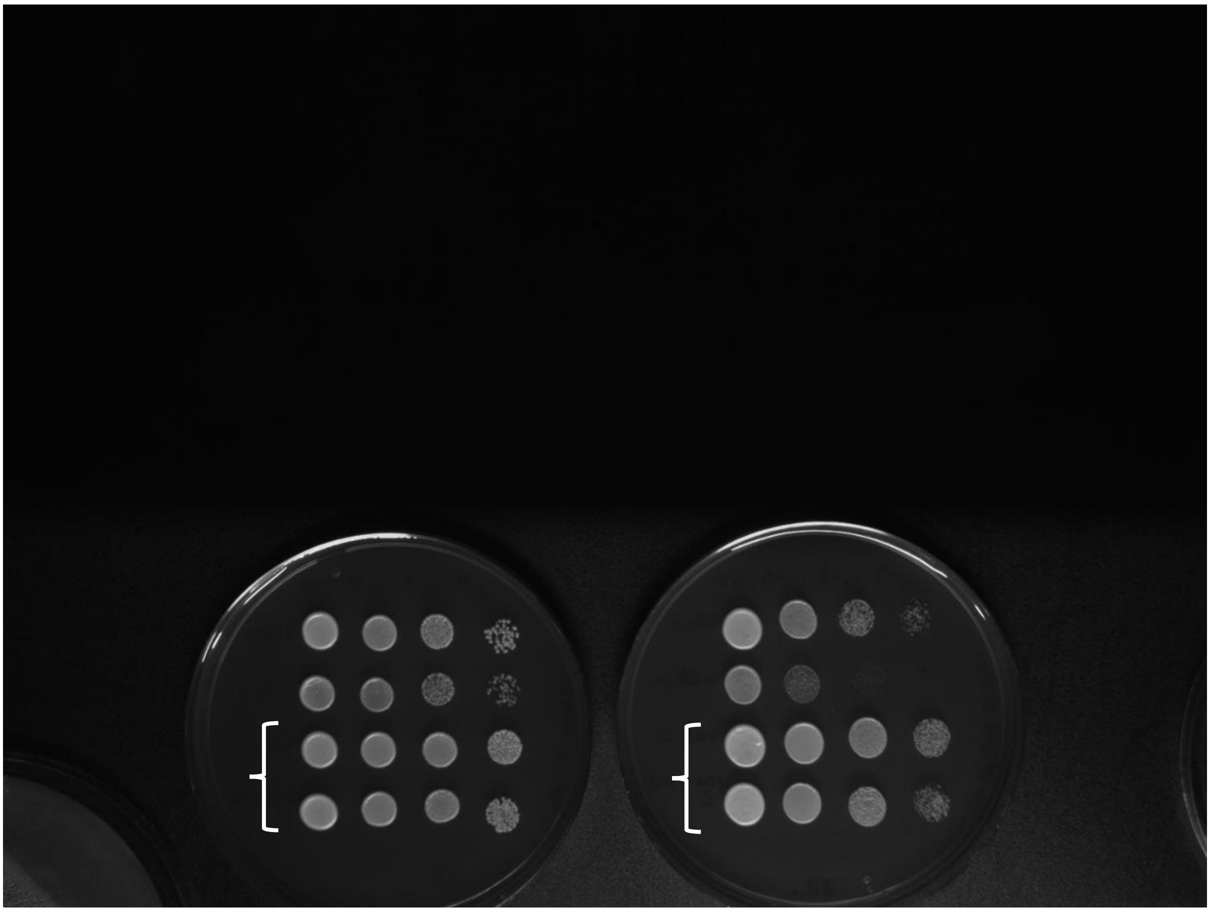

Figure 2D Lower Left panel

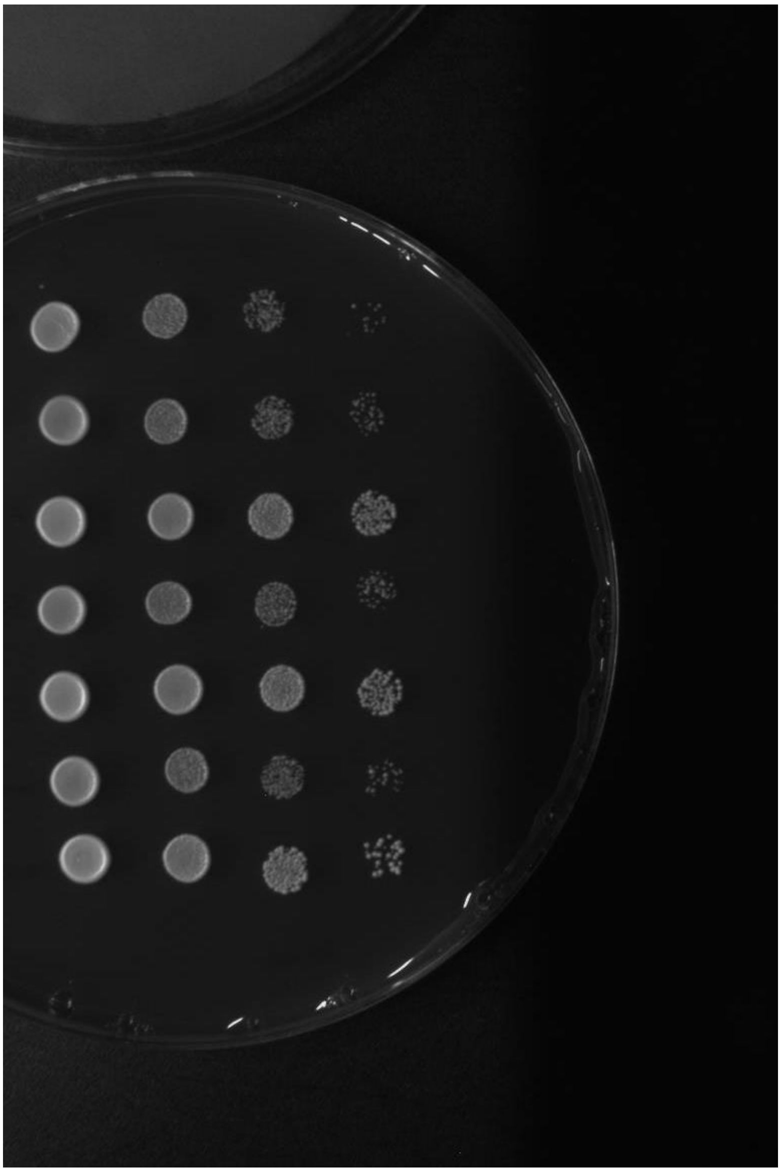

Figure 2D Lower Right panel

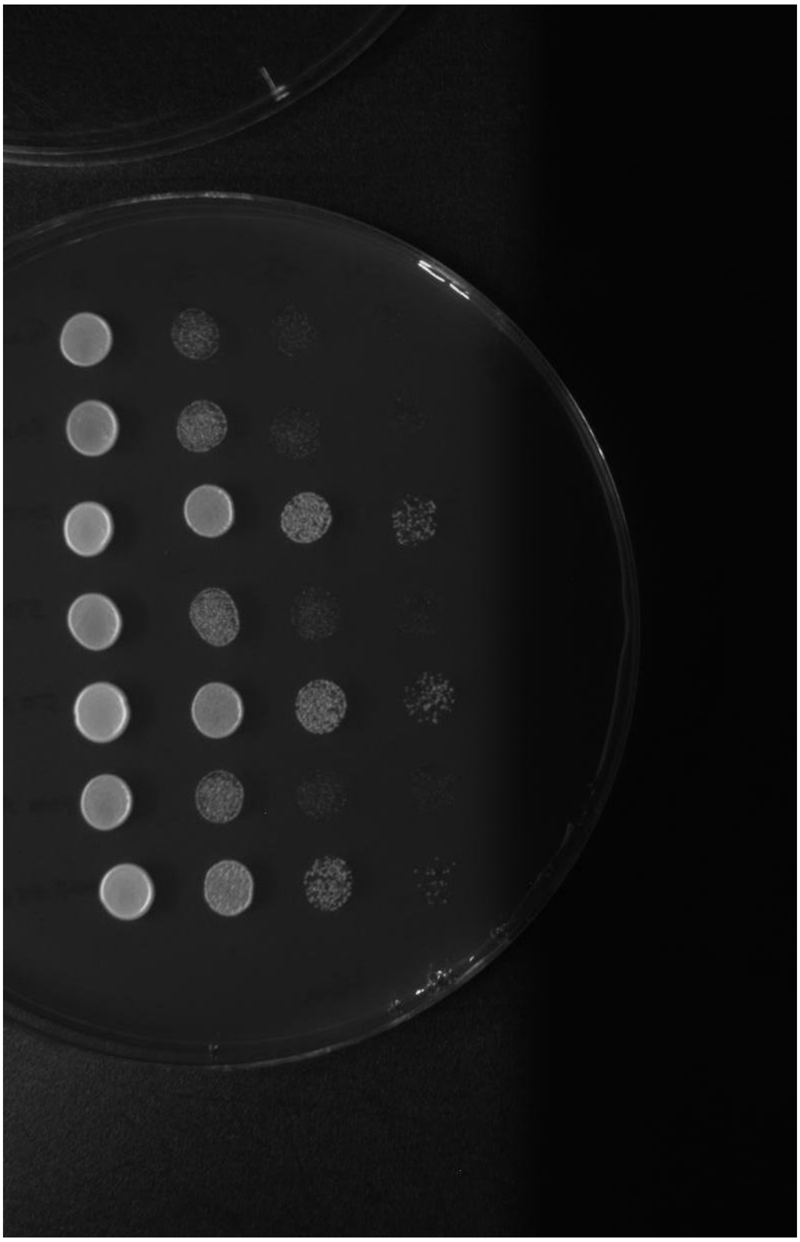

Figure 3A left panel

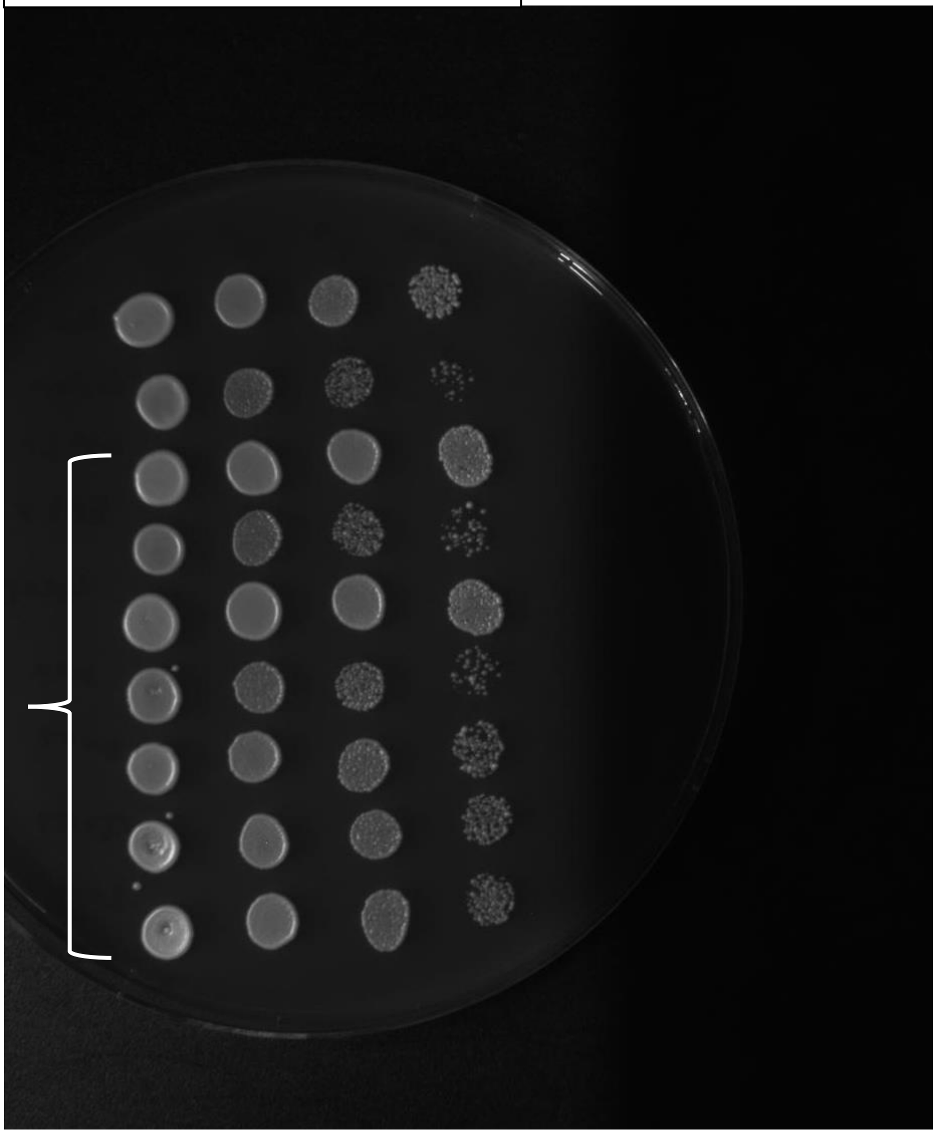

Figure 3A right panel

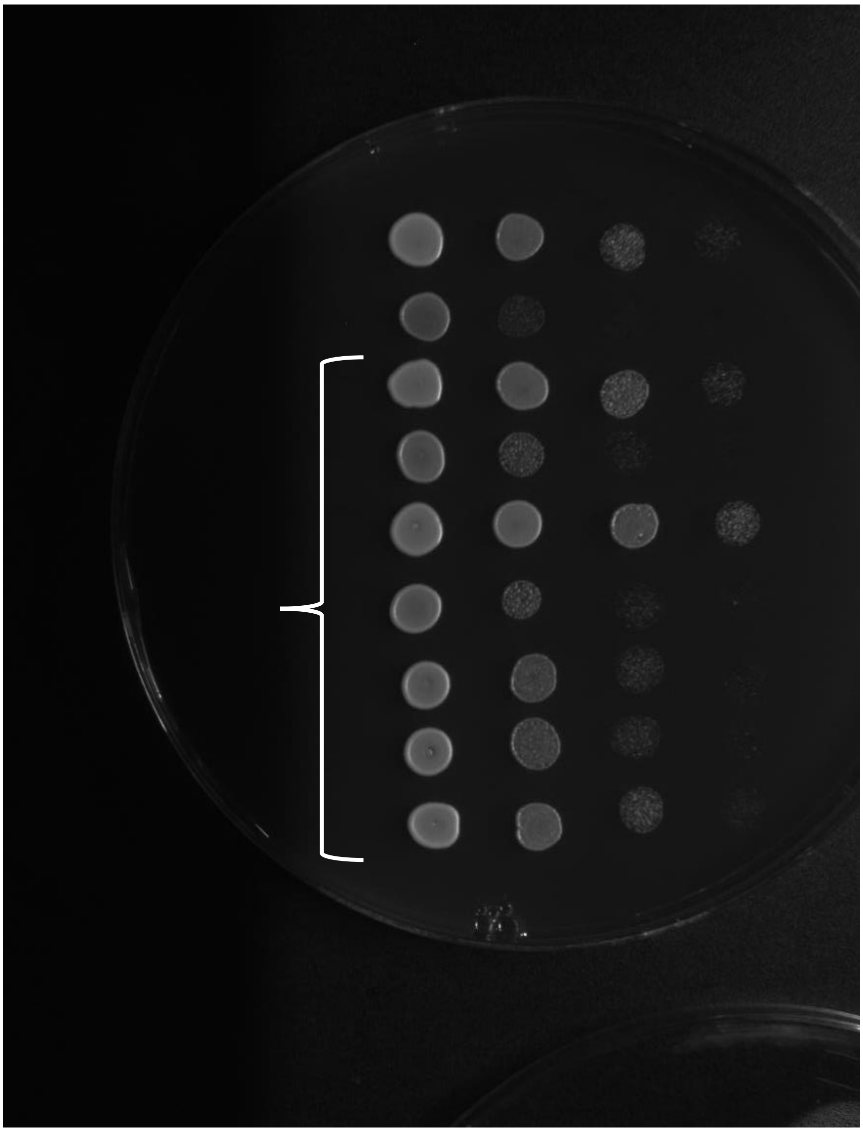

Supplement: S1 Data set — (ZIP) [file pone.0315052.s004.zip › minimal_data_set/Minimal_data_set_uncropped images_images.pdf]
