# Supplementary material for: Administration of novobiocin and apomorphine mitigates cholera toxin mediated cellular toxicity: Lessons from cholera toxin yeast model system
Source: PLoS One. 2024 Dec 5;19(12):e0315052. doi: 10.1371/journal.pone.0315052 (PMC11620602; doi:10.1371/journal.pone.0315052)
Supplement: Attachment — Submitted filename: PONE_reviewers comments _response 28.10.24.doc [file pone.0315052.s005.doc]

PONE-D-24-21529

Administration of novobiocin and apomorphine mitigates cholera toxin mediated cellular toxicity: Lessons from cholera toxin yeast model system

PLOS ONE

Dear Dr. Raychaudhuri,

Thank you for submitting your manuscript to PLOS ONE. After careful consideration, we feel that it has merit but does not fully meet PLOS ONE’s publication criteria as it currently stands. Therefore, we invite you to submit a revised version of the manuscript that addresses the points raised during the review process.


Please submit your revised manuscript by Nov 29 2024 11:59PM. If you will need more time than this to complete your revisions, please reply to this message or contact the journal office at [plosone@plos.org](mailto:plosone@plos.org). When you're ready to submit your revision, log on to https://www.editorialmanager.com/pone/ and select the 'Submissions Needing Revision' folder to locate your manuscript file.

Please include the following items when submitting your revised manuscript:

- A rebuttal letter that responds to each point raised by the academic editor and reviewer(s). You should upload this letter as a separate file labeled 'Response to Reviewers'.
- A marked-up copy of your manuscript that highlights changes made to the original version. You should upload this as a separate file labeled 'Revised Manuscript with Track Changes'.
- An unmarked version of your revised paper without tracked changes. You should upload this as a separate file labeled 'Manuscript'.

If you would like to make changes to your financial disclosure, please include your updated statement in your cover letter. Guidelines for resubmitting your figure files are available below the reviewer comments at the end of this letter.

If applicable, we recommend that you deposit your laboratory protocols in protocols.io to enhance the reproducibility of your results. Protocols.io assigns your protocol its own identifier (DOI) so that it can be cited independently in the future. For instructions see: [https://journals.plos.org/plosone/s/submission-guidelines#loc-laboratory-protocols](https://c05y1x9s.r.us-east-2.awstrack.me/L0/https:%2F%2Fjournals.plos.org%2Fplosone%2Fs%2Fsubmission-guidelines%23loc-laboratory-protocols/1/010f0192913ff0bd-b58d2119-4d2c-4e27-b355-bd6ac6e360c1-000000/PrIMS5ZB-q_YiY5El9ec8Of7WgQ=180). Additionally, PLOS ONE offers an option for publishing peer-reviewed Lab Protocol articles, which describe protocols hosted on protocols.io. Read more information on sharing protocols at [https://plos.org/protocols?utm_medium=editorial-email&utm_source=authorletters&utm_campaign=protocols](https://c05y1x9s.r.us-east-2.awstrack.me/L0/https:%2F%2Fplos.org%2Fprotocols%3Futm_medium=editorial-email%26utm_source=authorletters%26utm_campaign=protocols/1/010f0192913ff0bd-b58d2119-4d2c-4e27-b355-bd6ac6e360c1-000000/ErMMB_8RfJRHXvWJnE6HiFWarnU=180).

We look forward to receiving your revised manuscript.

Kind regards,

Rui Tada, Ph.D.
Academic Editor
PLOS ONE

Journal Requirements:

When submitting your revision, we need you to address these additional requirements.

1. Please ensure that your manuscript meets PLOS ONE's style requirements, including those for file naming. The PLOS ONE style templates can be found at 
https://journals.plos.org/plosone/s/file?id=wjVg/PLOSOne_formatting_sample_main_body.pdf and 
https://journals.plos.org/plosone/s/file?id=ba62/PLOSOne_formatting_sample_title_authors_affiliations.pdf

2. We note that the grant information you provided in the ‘Funding Information’ and ‘Financial Disclosure’ sections do not match.

When you resubmit, please ensure that you provide the correct grant numbers for the awards you received for your study in the ‘Funding Information’ section.

3. Thank you for stating the following in the Acknowledgments Section of your manuscript: 
We gratefully acknowledge Dr. Chetna Dureja for providing critical insights in this work. We
also acknowledge Dr. Deepak Sharma, Dr. Ravi Mishra and Dr. Ashwani Kumar for
providing small molecule library and cell culture facility. This work was partly supported by
grants from CSIR-IMTECH (OLP 151), CSIR-MLP39 and Science and Engineering
Research Board (CRG/2018/000297/SERB-GAP/0185). Sonali E Bhalerao and Himanshu
Sen, acknowledge Council of Scientific and Industrial Research (CSIR) for fellowships.
Sonali E Bhalerao also acknowledges CSIR-MLP39.

We note that you have provided funding information that is not currently declared in your Funding Statement. However, funding information should not appear in the Acknowledgments section or other areas of your manuscript. We will only publish funding information present in the Funding Statement section of the online submission form.

Please remove any funding-related text from the manuscript and let us know how you would like to update your Funding Statement. Currently, your Funding Statement reads as follows: 
 The author(s) received no specific funding for this work.

Please include your amended statements within your cover letter; we will change the online submission form on your behalf.

4. We note that your Data Availability Statement is currently as follows: All data are included in the manuscript.

Please confirm at this time whether or not your submission contains all raw data required to replicate the results of your study. Authors must share the “minimal data set” for their submission. PLOS defines the minimal data set to consist of the data required to replicate all study findings reported in the article, as well as related metadata and methods (https://journals.plos.org/plosone/s/data-availability#loc-minimal-data-set-definition).

For example, authors should submit the following data:

- The values behind the means, standard deviations and other measures reported;
- The values used to build graphs;
- The points extracted from images for analysis.

Authors do not need to submit their entire data set if only a portion of the data was used in the reported study.

If your submission does not contain these data, please either upload them as Supporting Information files or deposit them to a stable, public repository and provide us with the relevant URLs, DOIs, or accession numbers. For a list of recommended repositories, please see https://journals.plos.org/plosone/s/recommended-repositories.

If there are ethical or legal restrictions on sharing a de-identified data set, please explain them in detail (e.g., data contain potentially sensitive information, data are owned by a third-party organization, etc.) and who has imposed them (e.g., an ethics committee). Please also provide contact information for a data access committee, ethics committee, or other institutional body to which data requests may be sent. If data are owned by a third party, please indicate how others may request data access.

5. Please include captions for your Supporting Information files at the end of your manuscript, and update any in-text citations to match accordingly. Please see our Supporting Information guidelines for more information: http://journals.plos.org/plosone/s/supporting-information.

6. Please review your reference list to ensure that it is complete and correct. If you have cited papers that have been retracted, please include the rationale for doing so in the manuscript text, or remove these references and replace them with relevant current references. Any changes to the reference list should be mentioned in the rebuttal letter that accompanies your revised manuscript. If you need to cite a retracted article, indicate the article’s retracted status in the References list and also include a citation and full reference for the retraction notice.


[Note: HTML markup is below. Please do not edit.]

Reviewers' comments:

Reviewer's Responses to Questions

**Comments to the Author**

1. Is the manuscript technically sound, and do the data support the conclusions?

The manuscript must describe a technically sound piece of scientific research with data that supports the conclusions. Experiments must have been conducted rigorously, with appropriate controls, replication, and sample sizes. The conclusions must be drawn appropriately based on the data presented.

Reviewer #1: Yes

Reviewer #2: Yes

Reviewer #3: Yes

2. Has the statistical analysis been performed appropriately and rigorously?

Reviewer #1: No

Reviewer #2: Yes

Reviewer #3: Yes

3. Have the authors made all data underlying the findings in their manuscript fully available?

The [PLOS Data policy](http://c05y1x9s.r.us-east-2.awstrack.me/L0/http:%2F%2Fwww.plosone.org%2Fstatic%2Fpolicies.action%23sharing/1/010f0192913ff0bd-b58d2119-4d2c-4e27-b355-bd6ac6e360c1-000000/fXo0-LZVRUfhQvViV6mnhSOZHLY=180) requires authors to make all data underlying the findings described in their manuscript fully available without restriction, with rare exception (please refer to the Data Availability Statement in the manuscript PDF file). The data should be provided as part of the manuscript or its supporting information, or deposited to a public repository. For example, in addition to summary statistics, the data points behind means, medians and variance measures should be available. If there are restrictions on publicly sharing data—e.g. participant privacy or use of data from a third party—those must be specified.

Reviewer #1: Yes

Reviewer #2: Yes

Reviewer #3: Yes

4. Is the manuscript presented in an intelligible fashion and written in standard English?

PLOS ONE does not copyedit accepted manuscripts, so the language in submitted articles must be clear, correct, and unambiguous. Any typographical or grammatical errors should be corrected at revision, so please note any specific errors here.

Reviewer #1: Yes

Reviewer #2: Yes

Reviewer #3: Yes

5. Review Comments to the Author

Please use the space provided to explain your answers to the questions above. You may also include additional comments for the author, including concerns about dual publication, research ethics, or publication ethics. (Please upload your review as an attachment if it exceeds 20,000 characters)

Reviewer #1: The manuscript presents a study on the mitigation of cholera toxin (CTX) mediated cellular toxicity using novobiocin and apomorphine. Employing a yeast model system and subsequent validation in HT29 intestinal epithelial cells, the authors explore the efficacy of these compounds in reducing CTX-induced toxicity. The study is innovative, leveraging a high-throughput screening approach to identify potential therapeutic agents.

Strengths of the study:

• Innovative Use of Yeast Model: The use of S. cerevisiae as a model organism for screening small molecules is innovative and provides a high-throughput method for identifying potential therapeutic agents.
• Robust Screening Process: The screening of a diverse small molecule library led to the identification of promising compounds, showcasing a systematic approach to drug discovery.
• Relevance to Public Health: The study addresses a critical need for new cholera treatments, which could have significant implications for global health, particularly in cholera-endemic regions.

Limitations:

1. The study is limited to in vitro models, specifically yeast and HT29 cell lines. While these models are valuable for preliminary screening, the lack of in vivo data is a significant limitation. Animal studies are necessary to confirm the efficacy and safety of novobiocin and apomorphine in living organisms.

**Ans**: *We are in total agreement with the reviewer’s concern. Animal experiments will be conducted in collaboration with proper approval. In an animal model, the effect of apomorphine and novobiocin will also be examined in combination with new strategies. Recently, probiotic-mediated control of Vibrio cholerae has been shown quite effective in animal models (Sengupta et al., 2017; Nag et al., 2018). Similarly, L-ascorbic acid is also able to restrict the growth of Vibrio cholerae under in vitro conditions (Sen et al., 2024). So apomorphine and novobiocin will be examined either with the probiotic or in the presence of L-ascorbic acid. This demands extensive animal experimentation, and therefore, it will be taken up separately. Regarding safety, these are FDA-approved drugs. Apomorphine is still in use to treat Parkinson’s disease*. *It is interesting to note that some Parkinson’s drugs cause alteration of gut microbiota (van Kessel et al., 2022). This will also be checked in animal and fecal slurry models (Heer et al., 2024).*

**References:**

1. [*Sengupta C*](https://www.ncbi.nlm.nih.gov/pubmed/?term=Sengupta C%5BAuthor%5D&cauthor=true&cauthor_uid=28105081)*,* [*Ekka M*](https://www.ncbi.nlm.nih.gov/pubmed/?term=Ekka M%5BAuthor%5D&cauthor=true&cauthor_uid=28105081)*,* [*Arora S*](https://www.ncbi.nlm.nih.gov/pubmed/?term=Arora S%5BAuthor%5D&cauthor=true&cauthor_uid=28105081)*,* [*Dhaware PD*](https://www.ncbi.nlm.nih.gov/pubmed/?term=Dhaware PD%5BAuthor%5D&cauthor=true&cauthor_uid=28105081)*,* [*Chowdhury R*](https://www.ncbi.nlm.nih.gov/pubmed/?term=Chowdhury R%5BAuthor%5D&cauthor=true&cauthor_uid=28105081)*,* [*Raychaudhuri S*](https://www.ncbi.nlm.nih.gov/pubmed/?term=Raychaudhuri S%5BAuthor%5D&cauthor=true&cauthor_uid=28105081)*.Cross feeding of glucose metabolism byproducts of Escherichia coli human gut isolates and probiotic strains affect survival of Vibrio cholerae.* [*Gut Pathog.*](https://www.ncbi.nlm.nih.gov/pubmed/28105081) *2017 Jan 17;9:3. doi: 10.1186/s13099-016-0153-x. eCollection 2017.*
2. *Nag D, Breen P, Raychaudhuri S, Withey JH. Glucose metabolism by Escherichia coli inhibits* ***Vibrio cholerae*** *intestinal colonization of zebrafish. Infect Immun. 2018 Sep 24. pii: IAI.00486-18.  Dec; 86(12): e00486-18. doi: 10.1128/IAI.00486-18.*
3. *Sen, H, Kaur, M, Raychaudhuri S. L-Ascorbic acid restricts Vibrio cholerae survival in various growth conditions. Microorganisms 2024, 12, 492.* [*https://doi.org/10.3390/microorganisms12030492*](https://doi.org/10.3390/microorganisms12030492)
4. *van Kessel SP, Bullock A, van Dijk G, El Aidy S. Parkinson's Disease Medication Alters Small Intestinal Motility and Microbiota Composition in Healthy Rats. mSystems. 2022 Feb 22;7(1):e0119121. doi: 10.1128/msystems.01191-21.*
5. *Heer K, Kaur, M, Sidhu, D, Dey, P, Raychaudhuri, S. Modulation of gut microbiome in response to the combination of Escherichia coli Nissle 1917 and sugars: a pilot study using host-free system reflecting impact on interpersonal microbiome. Frontiers in Nutrition 2024****,*** *11, 1-17.* ***doi:*** *10.3389/fnut.2024.1452784*

2. The discussion section should include a comparison of the efficacy and potential advantages of novobiocin and apomorphine over existing cholera treatments and other emerging therapeutic approaches. This context would provide a clearer picture of the significance of the findings.

**Ans**: *The discussion section has been modified*.

3. Statistical significance analysis must be performed for all the graphs in the figures.

**Ans**: *We reanalyzed the data using different tools, but the significance of FACS analysis of percentage live cells is low. Kindly see.*

The manuscript is recommended for publication after addressing the limitations listed above.

Reviewer #2: Dear authors,

It is a good work that i think it contribute to the biological and toxicological sciences. I have one comment to improve your work as follows:

Please, prepare a more rigor and professional abstract showcasing your methodologies and results, and the contributions to the science.

**Ans:** *We have modified the sections as suggested.*

Reviewer #3: The resolution of figures seem very low. I could not figure out some of the labeling. I would increase the resolution and size accordingly. I was unable to verify the first two diagrams because of this issue.

**Ans**: *We have used the PLOS journals software for better resolution. Kindly see the new figures.*

6. PLOS authors have the option to publish the peer review history of their article ([what does this mean?](https://c05y1x9s.r.us-east-2.awstrack.me/L0/https:%2F%2Fjournals.plos.org%2Fplosone%2Fs%2Feditorial-and-peer-review-process%23loc-peer-review-history/1/010f0192913ff0bd-b58d2119-4d2c-4e27-b355-bd6ac6e360c1-000000/Z4j31ecLvF_Jfitfqm-W0HMnIks=180)). If published, this will include your full peer review and any attached files.


If you choose “no”, your identity will remain anonymous but your review may still be made public.


**Do you want your identity to be public for this peer review?** For information about this choice, including consent withdrawal, please see our [Privacy Policy](https://c05y1x9s.r.us-east-2.awstrack.me/L0/https:%2F%2Fwww.plos.org%2Fprivacy-policy/1/010f0192913ff0bd-b58d2119-4d2c-4e27-b355-bd6ac6e360c1-000000/zP5momS0XvT-3QLpzcIUca8JjKo=180).

Reviewer #1: No

Reviewer #2: **Yes:** Mohammad Amrollahi-Sharifabadi, Ph.D.

Reviewer #3: No
